# Physical Activity and Irritable Bowel Syndrome: The Role of Evolutionary Mismatch in Chronic Disease Risk

**DOI:** 10.1002/ajpa.70104

**Published:** 2025-08-01

**Authors:** Makenna B. Lenover Moyer, Mary K. Shenk

**Affiliations:** ^1^ Department of Anthropology The Pennsylvania State University, State College University Park Pennsylvania USA

**Keywords:** chronic illness, evolutionary medicine, exercise, irritable bowel syndrome, mismatch theory

## Abstract

**Objectives:**

Rising rates of noncommunicable diseases have been attributed to evolutionary mismatch between past physical activity and sedentary, post‐industrial behavior. Epidemiologic research suggests that sedentism increases irritable bowel syndrome (IBS) risk. We test this association with a population sample to assess whether physical activity mismatch is associated with IBS.

**Materials and Methods:**

This study recruited Pennsylvanians (age‐ and sex‐ matched to state population) to complete an online survey documenting digestion, demographics, and physical activity. IBS was diagnosed using Rome IV criteria, and data were analyzed using binary logistic regression.

**Results:**

The sample included 921 individuals (55.3% F; mean age = 38.78) with an IBS prevalence of 28.8%. Exercise vigor (none/low) was significantly associated with increased IBS risk [OR 1.469, 95% CI: 1.168–2.126, *p* = 0.0154], though other measures of exercise were not. BMI was a strong predictor of IBS continuously (sample mean BMI = 28.61) [OR 1.029, 95% CI: 1.011–1.048, *p* = 0.002], with higher BMI increasing IBS risk, especially for those overweight (25 < BMI < 30) [OR 1.734, 95% CI: 1.129–2.664, *p* = 0.012] or obese (BMI > 30) [OR 2.062, 95% CI: 1.361–3.125, *p* = 0.001]. BMI significantly mediated the relationship between exercise vigor and IBS.

**Discussion:**

Most research finds IBS is an illness driven by the environment, with exercise playing a protective role in disease risk. Our findings suggest that mismatch due to exercise levels alone is likely not a major driver of disease; instead, IBS may be driven by longitudinal effects of exercise (proxied here by BMI) alongside other environmental and behavioral factors contributing to energetic balance, such as diet and stress.

**Summary**

- Physical activity is not a major risk factor for IBS in a Pennsylvania population.
- BMI is significantly associated with IBS.
- BMI mediates the effects of exercise vigor on IBS, suggesting potential mismatch between present‐day environments and energy needs.

## 1 Introduction

The recent rise in noncommunicable diseases, especially in chronic diseases such as diabetes, hypertension, obesity, and cardiovascular disease, is the leading cause of death worldwide across countries of all income levels (Forouzanfar et al. 2016). A rich body of evolutionary medicine and public health work has demonstrated that the recent changes in behavior and lifestyle following industrialization are major contributors to this rise in chronic illness (Carrera‐Bastos et al. 2011; Clatici et al. 2018). One behavior associated with the prevalence and severity of these chronic illnesses is physical inactivity (Forouzanfar et al. 2016).

Examining the evolution of behavior over time, we see physical activity levels decrease due to changing economic and production environments. While past and present‐day hunter‐gatherers exerted large amounts of energy for food production, post‐industrial societies are much more sedentary (Pontzer et al. 2012). Food acquisition strategies were a main driver of activity levels in the past, requiring physiological adaptations for high levels of endurance, needed for activities including walking, running, and digging (Lieberman 2015). That being said, as expected by evolutionary theory, past humans likely avoided excess energy exertion in nutritionally precarious environments, only moving as much as they needed to in order to meet caloric intake and maintain energy for reproduction (Lieberman 2015). This resulted in an energetic balance between physical movement and caloric intake based on subsistence‐focused activities like walking long distances or foraging, a balance that is still predominant in today's hunter‐gatherers (Lieberman 2015).

Until recently, humans generally lacked the technological and socioenvironmental conditions to be both sedentary and in a calorie excess. These conditions have become commonplace with industrialization, however, disrupting our evolved energetic balance with the potential for health consequences. Beyond the short term and acute effects of exercise, such as energy expenditure, the longitudinal effects of physical activity have long‐term physiological health implications, such as decreased muscle mass and strength, reduced cardiovascular health and dysfunction, loss of bone mass, increased visceral fat, and even negative neurologic impacts on cognitive function (Pinto et al. 2023; Thyfault et al. 2015). This perspective falls within the evolutionary framework of mismatch, that health problems may arise because our evolved biology is not adapted to present‐day environments. Many illnesses have been studied as arising from mismatch attributable to reduced physical activity, including diabetes and heart disease (Aune et al. 2015; Perry et al. 2023). Other illnesses may be suited for a mismatch‐based explanation, but there is not yet enough evidence available to draw these conclusions. One such illness is irritable bowel syndrome (IBS).

IBS is a disorder of gut‐brain interaction characterized by a combination of chronic or periodically recurring symptoms, including bloating, constipation, diarrhea, and abdominal pain not linked to other biochemical or structural abnormalities of the GI tract (Enck et al. 2016; Manning et al. 1978; Weaver et al. 2017). The illness is complex in both symptom manifestation and treatment response; patients may experience a multitude of individually variable digestive symptoms, and treatment success rates are also highly patient‐specific (Weaver et al. 2017). IBS is a global phenomenon, with varying worldwide prevalence ranging 6%–40%, averaging 11.2% globally, and affecting a reported 15%–20% of the United States population. In addition, rates are rising, and it is suspected that many people remain undiagnosed, with actual rates nearing 30% (Sperber et al. 2012; Defrees and Bailey 2017). Patterns of global prevalence are consistent with those we would expect if IBS were driven by mismatch, with rates highest in post‐industrial countries, lowest in non‐industrial countries, and rising rapidly in countries undergoing industrial/market transitions. Our recent review article details the potential mechanistic pathways by which mismatch may lead to IBS, including digestion and diet (Carrera‐Bastos et al. 2011; Trakman et al. 2022), modified hygiene environments and immune system dysregulation (Rook 2009; Rook et al. 2013), and an impaired brain‐gut axis driven by mental health and as well as changes in behaviors including sleep and exercise (Fichna and Storr 2012; Lenover and Shenk 2023), the latter of which we examine here.

Little is known about the underlying etiology of IBS (Fichna and Storr 2012; Trakman et al. 2022). Genetic research is still underway, but current studies find a complex, multigenic gene–environment interaction that features a minimal genetic contribution alongside a significant attribution to behavior and environment (Lembo et al. 2007; Mahurkar‐Joshi and Chang 2020; Stilling et al. 2014; Talley 2005). This suggests IBS has an epigenetic component, likely driven by behavioral risk factors including diet, hygiene, stress, and sleep quality, all of which contribute dynamically to individual disease susceptibility (Lenover and Shenk 2023). Another, separate causal trajectory of IBS may be post‐infectious in origin, resulting as a downstream health concern from bacterial gastrointestinal illnesses (Dunlop et al. 2003).

Limited existing evidence studying physical activity levels in relation to IBS demonstrates a potential link between physical activity levels and disease. Several cross‐sectional and case–control studies have found that a sedentary lifestyle is positively correlated with IBS in countries undergoing the industrial transition, including India, China, and Saudi Arabia (Aljammaz et al. 2020; Guo et al. 2015; Khanna et al. 2021). In China, for example, physical inactivity was associated with a nearly 3.6 times higher odds of having IBS, and in India, of 300 IBS patients studied, only 13% met or exceeded recommended physical activity levels (Guo et al. 2015; Khanna et al. 2021). Additionally, both medical and undergraduate students who report low levels of physical activity in Peru, Japan, Saudi Arabia, and Nigeria all demonstrated a significantly higher likelihood of developing IBS than their physically active peers (AlButaysh et al. 2020; Vasquez‐Rios et al. 2019; Wani et al. 2020; Yamamoto et al. 2023). Yet, since these countries are largely undergoing the industrial transition and the study population samples are often homogenous (i.e., students, IBS patients), these studies have limited generalizability to diverse populations in industrialized contexts (Lenover and Shenk 2023). However, new prospective cohort studies conducted in the United Kingdom on UK Biobank participants reaffirm these findings in the context of an industrialized country. Physical activity was identified to be a protective factor against IBS risk over the course of 12.6 years, and low levels of physical activity were found to have adverse effects on digestive health (Ho et al. 2024; Gao et al. 2024; Wu et al. 2023).

Additional evidence for the role of exercise in IBS risk comes from a small selection of literature demonstrating the effectiveness of exercise‐based interventions on IBS. Yoga is one potential IBS treatment, found to be as effective in treating symptoms as dietary changes (Schumann et al. 2018). Another study examined the long‐term impact of increased physical activity and IBS and found that sustained physical activity, across an average span of 5 years, contributed to long‐term relief of IBS symptoms, both digestive and associated psychological symptoms (Johannesson et al. 2015). While these studies in part signify a relationship between intervention and disease outcome, it is important to apply caution when hypothesizing the strength of disease causality, as most research has been conducted on clinical samples without control groups, limiting our interpretation of exercise as a preventative measure for IBS in the context of the broader population (Lenover and Shenk 2023). Moreover, exercise is also associated with improving stress levels, and stress is another known risk factor for IBS (Malan‐Muller et al. 2022; Remes‐Troche et al. 2015; Vasquez‐Rios et al. 2019); thus, physical activity may simply be improving symptoms through this pathway, rather than independently.

Work is thus still needed to determine the relationship between exercise and IBS risk, especially since one study found that IBS prevalence rates among endurance athletes were comparable to the general population (Killian and Lee 2019). More information regarding physical activity level is needed to conclude whether sedentism is a significant risk factor for disease across populations, and additional studies are needed investigating different types of physical activity and symptom relief to distinguish what types of exercise may improve symptoms and which may exacerbate them. It is unclear how much physical inactivity increases the risk of IBS and whether this is a dose–response effect with a maximum threshold. Given that existing evidence suggests both physical inactivity as a risk factor and intense endurance activities as a risk factor, it is possible that energy needs for digestive maintenance fall within a range that was met in the human past but is now disrupted.

This study uses cross‐sectional survey data collected from a diverse population sample within Pennsylvania, United States to determine the relationship between exercise and IBS risk. We collect data on the types of exercise practiced, levels of physical activity (including at place of work and outside employment), and body mass index (BMI) to assess physical activity across the life course. Through this, our study begins to differentiate specific physical activity‐related risk factors and situate these in the context of IBS and digestive health. This is a key step in better understanding the energetic tradeoffs and evolutionary mismatch between post‐industrial environments and digestive health, particularly to evaluate if physical activity‐related mismatch is associated with IBS as it is with other illnesses, including diabetes, obesity, and heart disease. Additionally, these results may inform IBS treatments, clinical research, and preventative interventions promoting physical activity to reduce rates of digestive chronic disease.

## 2 Materials and Methods

### 2.1 Study Design and Recruitment

We conducted a quantitative, cross‐sectional online survey study with participants recruited via quota sampling, collecting information about demographics, digestive health, lifestyle, and physical activity. To establish our sample in Pennsylvania, potential participants were recruited using Prolific, an online survey distribution tool that collects responses from verified individuals who fit within appropriate inclusion criteria for each study (Prolific 2014). Prolific provides a transparent, accountable, and reliable method of crowdsourcing research participants and has been used increasingly frequently by survey researchers due to its participant quality and ease of recruitment (Palan and Schitter 2018). Several studies have investigated the data quality of responses recruited using Prolific in comparison to other recruitment sites, such as Qualtrics and MTurk, and consistently found Prolific resulted in the highest quality data, including in the categories of comprehension, honesty, and attention (Douglas et al. 2023; Peer et al. 2022). Prolific respondents also reported taking the fewest online surveys in comparison to other recruitment platforms (Peer et al. 2017).

To ensure diversity of sample characteristics and representativeness of the sample for a variety of life experiences, we used a quota sampling method that replicated the age and sex composition of the State of Pennsylvania (United States Census Bureau 2023). This sampling method was crucial for the study to generalize to a real‐world population of variable individuals with and without IBS, and to avoid common sampling concerns that emerge when sampling via online recruitment, such as over‐ or under‐representation of people across demographic characteristics including sex or age. Participants were compensated $7 for their participation. Approval for this study was obtained from The Pennsylvania State University Institutional Review Board (IRB) (Project ID: STUDY00020610). Participants were asked to give consent via an online form prior to data collection.

### 2.2 Participants & Study Power Analysis

A total of 1026 individuals began the survey, and 921 individuals completed the survey (89.8% completion rate, a high rate for this type of research). Only two participants failed the attention checks and were dropped from the sample, while the other individuals began but failed to complete the survey. The inclusion criteria for participants were (a) living in Pennsylvania at the time of study, (b) between the ages of 20 and 65, (c) born in and grew up in the United States, and (d) having a willingness to discuss personal details about digestive health and bowel movements. People who had an official medical diagnosis of inflammatory bowel disease (either ulcerative colitis or Crohn's disease) were excluded from the study since these conditions could cause potential inaccuracies with IBS diagnosis.

This sample provided needed power for all analyses; pre‐hoc power calculations (0.05 alpha and 0.20 beta) conducted before data collection began based on available data from previous studies estimated needing a sample ranging from 82 to 306 individuals for the variables of interest in this paper (Table S1).

### 2.3 Instrumentation

The survey instrumentation was designed to acquire the participant's IBS disease status, demographic information, and exercise/physical activity patterns, both recreational and during the workday. IBS was diagnosed using diagnostic criteria‐based questions, the Rome IV criteria diagnostic questionnaire for IBS, which assigns people to either having or not having IBS. Diagnosis is based on a series of questions regarding stool frequency, stool form, and number of days with abdominal pain (Drossman and Hasler 2016). Physicians use the Rome IV diagnostic criteria when diagnosing patients, but the same questions can be implemented in a straightforward way in questionnaire research. Indeed, the Rome IV criteria were originally developed to standardize gastroenterological research, and its usage in survey studies is not only standard but regarded as best practice to ascertain comparable prevalence rates (Schmulson and Drossman 2017). Not all people with IBS have been officially diagnosed by a doctor (Defrees and Bailey 2017; Killian and Lee 2019), so this criteria approach also allows for the study of individuals experiencing this syndrome regardless of structural health access limitations and diagnosis biases. Participants were also asked if they had received a physician diagnosis of IBS.

Basic demographic and control variable data were collected, including socioeconomic status, employment, racial identity, and both gender and sex assigned at birth. Height and weight were also collected, and BMI was calculated. The survey then asked a series of quantitative questions about participants' lifestyles, collecting variables related to recreational and work‐based exercise practices. The existing literature was used to develop those survey questions, which then underwent extensive pre‐testing and pilot data collection. The questionnaire included overall daily physical activity levels in the past 3 months, the average time spent per week exercising, and the vigor of that activity, and specific types of physical activities done in employment settings and during elected exercise (Appendix I: Survey Instrument). These questions were informed by CDC standards for healthy levels of weekly physical activity, either 150 min of moderate aerobic intensity exercise or 75 min of vigorous intensity exercise per week (CDC 2022).

Study data were collected and managed using REDCap electronic data capture tools hosted at Penn State Health Milton S. Hershey Medical Center and Penn State College of Medicine (Harris et al. 2009). REDCap (Research Electronic Data Capture) is a secure, web‐based application designed to support data capture for research studies.

### 2.4 Statistical Analysis

Data were examined to evaluate predicted relationships between exercise variables and IBS outcomes. While there were cases of missing data (< 3%), these were random, with no patterns related to any specific demographics or questions, as well as being independent of IBS status. Given the limited scope of missingness, no data imputation was conducted for missing values.

We used binary logistic regression to test our predictions. All analyses were completed using R Statistical Software (version 4.3.0) (R Core Team, 2021). Continuous age and BMI variables were centered around the mean (age = 38.78, BMI = 28.67). The outcome variable for all predictions remains the same: Rome IBS (“1”) or no Rome IBS (“0”). Rome IBS status was chosen for all analyses as this is the standard in IBS clinical research and captures active IBS status at the time of data collection, which is particularly meaningful for drawing conclusions about the relationship between current behaviors and IBS. Rome IBS status also captures all cases of IBS regardless of whether they have been formally diagnosed, thus presenting a more complete understanding of the disease. Results of the physician‐diagnosis analyses are available in the supplemental materials.

We tested the bivariate relationships between individual exercise variables (ordinal scales as reported on the survey) and IBS status, controlling for age, sex, and race. Interaction effects between these variables and controls were also explored. We next sought to understand the effect of BMI on all significant exercise predictors, assessing correlation via point‐biserial correlation coefficient, testing BMI both as a co‐regressor and as a moderator via interaction terms, and conducting a causal mediation analysis with nonparametric bootstrapping, based on Hayes' approach (Hayes 2009).

To assess the predictive power of exercise levels on IBS status, data reduction by model selection methods derived from likelihood theory were used to determine the most representative factors in a stepwise fashion. Systematic combinations were drawn using the R package glmulti, and the model with the lowest Akaike information criterion (AIC) was selected. This model was tested for discrimination by creating a receiver operating characteristic (ROC) curve and calculating the area under the curve (AUC), reported as a concordance statistic (C‐statistic) for binary models. Calibration was also assessed using calibration‐in‐the‐large and calibration slope to evaluate the model's predictive performance. Finally, predictive ability was assessed via a 10‐fold cross‐validation, evaluated using three metrics: Root Mean Squared Error (RMSE), R‐squared, and Mean Absolute Error (MAE).

## 3 Results

### 3.1 Sample Characteristics & Demographic Trends

Responses were collected between November 2023 and October 2024. The sample had an IBS prevalence of 28.77% (*n* = 265), as diagnosed via the Rome IV criteria, while 16.61% (*n* = 153) of the sample had received a physician diagnosis of IBS. This allowed us to capture individuals who were currently symptomatic, diagnosed by a practitioner and not symptomatic, and those who were both symptomatic and diagnosed via a physician (Table 1). This finding aligned with predictions that IBS is underdiagnosed in clinical settings. Those with a Rome IBS classification, as this is the diagnostic used by gastroenterologists and provides clear comparability of diagnosis across respondents, were used in the main analysis (Table 2, Table S2), though those with a physician diagnosis were also explored secondarily (Tables S3, S5, S6).

**TABLE 1:** IBS status as diagnosed via Rome criteria and participant reported physician diagnosis history.

	Rome IV diagnosis	No Rome IV diagnosis
Physician Diagnosis	92	60
No Physician Diagnosis	173	596

**TABLE 2:** Characteristics of study participants with Rome IV diagnosed IBS and healthy controls.

Characteristic	Participants with IBS (*n* = 265)	Participants without IBS (*n* = 656)
Age (Mean ± SD)	38.12 ± 10.91	39.04 ± 12.32
Sex (*n*, % Female)	183 (69.05%)	326 (49.69%)
IBS Subtype—*n* (%)		
IBS‐Diarrhea	84 (31.69%)	NA
IBS‐Constipation	98 (36.98%)	
IBS Mixed	83 (31.32%)	
Region of Residence—*n* (%)		
Rural	62 (23.39%)	147 (22.41%)
Suburban	142 (53.58%)	352 (53.66%)
Urban	60 (22.64%)	151 (23.02%)
Race/ethnicity—*n* (%) *Multiple selections allowed*		
Asian or Pacific Islander	4 (1.50%)	25 (3.81%)
Black or African American	33 (12.45%)	96 (14.63%)
Hispanic or Latino	7 (2.64%)	21 (3.20%)
Native American or Alaskan	1 (0.40%)	4 (0.61%)
Native White or Caucasian	237 (89.43%)	539 (82.16%)
Body Mass Index (BMI) (Mean ± SD)	30.00 ± 8.50	28.12 ± 7.51
Overall Sedentary Activity Level—*n* (%)	68 (25.66%)	126 (19.21%)
1 or Fewer Hours of Weekly Physical Activity—*n* (%)	63 (23.77%)	139 (21.20%)
Mild or No Additional Weekly Exercise—*n* (%)	179 (67.55%)	367 (55.95%)

The sample was 55.26% female (*n* = 509). This sample closely mimics the age and sex distribution of the population of Pennsylvania, achieved by quota sampling with these proportions in mind. The race and ethnicity breakdown of the sample also closely matches the larger Pennsylvania population: 80.6% of the Pennsylvania population identifies as white and 84.26% of our sample identifies as white; 22% of the Pennsylvanian population lived in rural areas in 2020, as did 22.69% of our sample (United States Census Bureau 2023; Center for Rural PA 2020). Our sample also aligns with state BMI averages; 33.3% of Pennsylvanians have a BMI of 30 or over (classified as obese) while 33.65% of this sample reported a BMI over 30 (United Health Foundation 2023).

The relationship between demographic variables for the sample and controls for modeling was investigated via binary logistic regression (Table 3, Table S4). Region of residence, age, and income level were not significant predictors of IBS outcomes. Sex and race are statistically significant predictors of IBS status, with females [OR: 2.238, 95% CI: 1.653–3.032] and white individuals [OR: 1.744, 95% CI: 1.129–2.694] having a higher risk of IBS.

**TABLE 3:** Model outputs for demographic variables and controls in relation to Rome IV diagnosed IBS.

Model	Sample size	Coefficient estimate	Odds ratio	Confidence interval	*p*	AIC
IBS ~ Age						1096
Age	906	−0.0064	0.994	0.982–1.006	0.304	
IBS ~ Race						1104
Not White	144					
White	776	0.5560	1.744	1.129–2.694	0.012*	
IBS ~ Sex						1074
Male	404					
Female	509	0.8058	2.238	1.653–3.032	1.95e−7 ***	
IBS ~ Age + Sex + Race	899					1054
Age		−0.0122	0.988	0.657–2.749	0.050	
Sex		0.8456	2.329	1.508–3.599	8.95e−8***	
Race		0.5764	1.779	1.152–2.749	0.012*	

*Note:* *(0.01 < *p* ≤ 0.05); ***(*p* ≤ 0.001).

### 3.2 Individual Relationships Between Exercise Variables and IBS

The relationships between individual, self‐reported exercise variables and IBS were investigated via binary logistic regression. Self‐reported levels of daily physical activity (sedentary, moderately active, or vigorously active/extremely active) were not significantly related to IBS outcome (Table 4); neither was weekly time spent engaging in physical activity (over/under 75 min, over/under 150 min) (Table 4).

**TABLE 4:** Model outputs for individual exercise and BMI variables and controls in relation to Rome IV diagnosed IBS.

Model	Sample size	Coefficient estimate	Odds ratio	95% confidence interval	*p*	AIC
IBS ~ Overall Physical Activity + Controls						1044
*Vigorous/Extremely Active*	140					
Sedentary	191	0.3061	1.358	0.353–4.890	0.231	
Moderately Active	553	0.0074	1.007	0.629–5.665	0.974	
Age		−0.0129	0.987	0.971–1.032	0.047*	
Sex		0.7943	2.213	0.866–3.844	8.81e−7***	
Race		0.5909	1.806	1.231–2.864	0.011*	
IBS ~ Over 75 min of Activity a Week + Controls						1046
*Over 75 min of Activity*	589					
Under 75 min of Activity	308	0.1618	1.176	0.863–1.602	0.306	
Age		−0.0129	0.987	0.975–0.999	0.045*	
Sex		0.8304	2.294	1.677–3.138	2.01e−7***	
Race		0.565	1.759	1.113–2.768	0.015*	
IBS ~ Over 150 min of Activity a Week + Controls						1046
*Over 150 min of Activity*	412					
Under 150 min of Activity	476	−0.1478	1.759	0.639–1.164	0.334	
Age		−0.0129	0.863	0.975–0.999	0.045*	
Sex		0.8333	0.987	1.683–3.146	1.75e−7***	
Race		0.5647	2.301	1.117–2.768	0.015*	
IBS ~ Vigor of Activity + Controls						1046
*Moderate/High Vigor*	360					
None/Mild Vigor	533	0.3844	1.469	1.076–2.004	0.015*	
Age		−0.0133	0.987	0.974–0.999	0.041*	
Sex		0.8006	2.227	1.629–3.045	5.32e−7***	
Race		0.5550	1.742	1.109–2.737	0.016*	
IBS ~ Meeting CDC Activity Levels + Controls						1045
*Meeting CDC Levels*	336					
Not Meeting CDC Levels	553	0.3083	1.361	0.994–1.101	0.055	
Age		−0.0135	0.987	0.974–0.999	0.038*	
Sex		0.8176	2.265	1.656–3.098	3.12e−7***	
Race		0.5483	1.730	1.101–2.720	0.018*	

*Note:* *(0.01 < *p* ≤ 0.05); ***(*p* ≤ 0.001).

Vigor of weekly physical activity, dichotomized for statistical power (none/mild or moderate/high intensity) was a predictor of IBS, with those engaging in no or low vigor activity having 1.469 times higher odds of having IBS than those engaged in moderate or high vigor activities [95% CI: 1.168–2.126, *p* = 0.015] (Table 4). According to the CDC, a healthy amount of physical activity per week is either 150 min of moderate vigor or 75 min of high vigor activity (CDC 2022). While vigor was a significant predictor in isolation, when combined with time spent on exercise based on CDC recommendations, it was no longer significant (Table 4).

### 3.3 BMI, Physical Activity, and IBS

BMI was calculated via reported height and weight of participants and investigated from a variety of angles. BMI as a continuous variable (mean‐centered at 0) was a significant predictor of IBS, with higher BMI increasing risk (Table 5) (CDC 2022). When analyzed as the three CDC weight categories, underweight (BMI < 18.5)/healthy weight (18.5 < BMI < 25), overweight (25 < BMI < 30), and obesity (BMI > 30), overweight and obesity were significant predictors of IBS (Table 5) (CDC 2022).

**TABLE 5:** Model outputs for BMI and controls in relation to Rome IV diagnosed IBS.

Model	Sample Size	Coefficient Estimate	Odds Ratio	95% Confidence Interval	*p*	AIC
IBS ~ BMI Continuous + Age + Sex + Race						1041
BMI	818	0.02931	1.029	1.011–1.048	0.002**	
Age		−0.0151	0.985	0.972–0.997	0.022*	
Sex		−0.8112	2.251	1.646–3.078	3.8e−7***	
Race		0.5824	1.808	1.145—	0.011*	
IBS ~ 3 Scale BMI + Sex + Age + Race						1042
*Underweight (BMI < 18.5)/Healthy (18.5 < BMI < 25)*	312					
Overweight (25 < BMI < 30)	276	0.5508	1.734	1.129–2.664	0.012*	
Obese (BMI > 30)	310	0.7236	2.062	1.361–3.125	0.001***	
Age		−0.0209	0.979	0.965–0.994	0.005**	
Sex		0.935	2.458	1.697–3.291	2.57e−7***	
Race		0.5140	1.672	1.021–2.737	0.041*	

*Note:* *(0.01 < *p* ≤ 0.05); **(0.001 < *p* ≤ 0.01); ***(*p* ≤ 0.001).

While exercise vigor was predictive of IBS independently, we tested the potential mediating or confounding role of BMI on this relationship. The two were weakly correlated (correlation coefficient *r*_*pb*_ = −0.185), and when BMI was treated as a covariate with exercise vigor, exercise vigor was no longer a significant predictor. The interaction between BMI and vigor was also insignificant, suggesting no moderating role.

**FIGURE 1:**
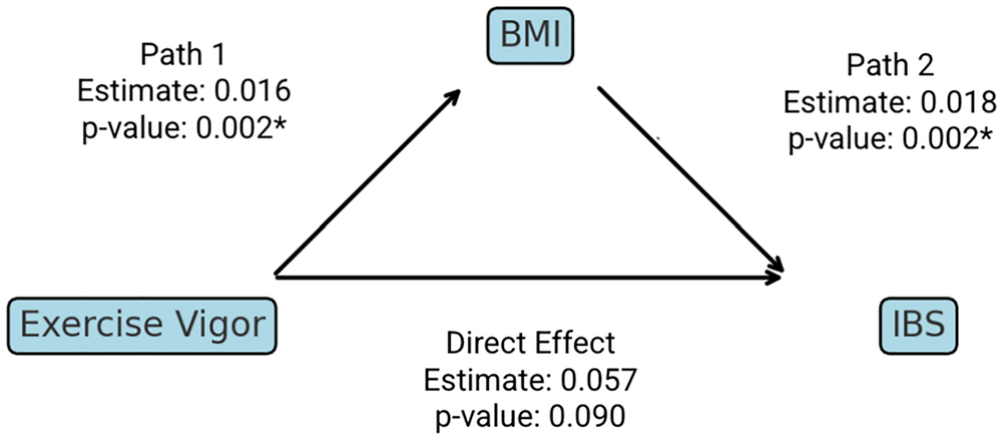
Mediation analysis depicting both the direct and indirect pathways from exercise vigor to IBS with BMI as a mediator.

In our mediation analysis, however, we found that BMI significantly mediates the relationship between exercise vigor and IBS risk (Table 6, Figure 1). The average causal mediation effect (ACME) was significant (*p* = 0.002), suggesting an indirect effect through BMI, while the average direct effect (ADE) was not significant (*p* = 0.09). These results indicate that exercise vigor does not independently associate with IBS risk apart from its influence on BMI. The proportion of the total effect mediated by BMI was approximately 22% (*p* = 0.022). While the cross‐sectional nature of our data limits the ability to definitively establish temporal precedence, the hypothesized mediation pathway aligns with existing evidence that exercise influences BMI, which in turn affects IBS risk. Future longitudinal studies are needed to confirm these findings.

**TABLE 6:** Results of BMI mediation analysis.

Effect	Estimate	95% CI lower	95% CI upper	*p*
ACME (control)	0.01573	0.00407	0.03000	0.002**
ACME (treated)	0.01787	0.00491	0.03000	0.002**
ADE (control)	0.05636	−0.00859	0.12000	0.090
ADE (treated)	0.05850	−0.00898	0.13000	0.090
Total Effect	0.07423	0.01322	0.14000	0.020*
Prop. Mediated (control)	0.21189	0.03268	1.21000	0.022*
Prop. Mediated (treated)	0.24073	0.03899	1.20000	0.022*
ACME (average)	0.01680	0.00445	0.03000	0.002**
ADE (average)	0.05743	−0.00879	0.12000	0.090
Prop. Mediated (average)	0.22631	0.03582	1.21000	0.022*

*Note:* *(0.01 < *p* ≤ 0.05); **(0.001 < *p* ≤ 0.01).

### 3.4 Overall IBS Predictive Model

Using stepwise model selection methods based on AIC criteria suggested that the best model for IBS included continuous BMI, exercise vigor, sex, age, and race. Yet the discrimination analysis showed this model had low predictive power (C‐statistic = 0.6495, 95% CI: 0.6136–0.7002) (Table 7). Calibration of the model was assessed using both calibration‐in‐the‐large and calibration slope methods. The calibration‐in‐the‐large showed a significant intercept estimate of 0.38957 (*p* < 0.001), suggesting this model tends to overestimate the likelihood of the outcome. This is further supported by the significant calibration slope estimate of 4.8901 (*p* < 0.001), indicating the model predictions are overconfident. Finally, the results of a 10‐fold cross‐validated generalized linear model further reaffirm the low predictive ability of this model (RMSE = 0.4452, *r* ^2^ = 0.0504, MAE = 0.3952).

**TABLE 7:** Model selection output for best fit model for IBS within the study population.

Model	Coefficient Estimate	Odds Ratio	95% Confidence Interval	*p*	AIC
IBS ~ Vigor + BMI Continuous + Age + Sex + Race					1032
Vigor	0.2735	1.315	0.954–1.811	0.094	
BMI	0.0255	1.026	1.006–1.045	0.008**	
Age	−0.0163	0.983	0.971–0.997	0.014*	
Sex	0.7764	2.174	1.585–2.981	1.45e−6***	
Race	0.5643	1.758	1.111–2.781	0.016*	

*Note:* *(0.01 < *p* ≤ 0.05); **(0.001 < *p* ≤ 0.01); ***(*p* ≤ 0.001).

## 4 Discussion

This study set out to understand if the environmental mismatch between physical activity levels in post‐industrial environments and evolved energy expenditure needs is associated with IBS. While this pattern has been demonstrated for other chronic illnesses, including diabetes and obesity, no studies have investigated the effects of exercise in the United States on IBS despite emerging epidemiological evidence that lowered physical activity levels in industrializing countries are associated with IBS, as well as emerging prospective evidence in the United Kingdom showing the same trends (Perry et al. 2023; Vasquez‐Rios et al. 2019; Wani et al. 2020; Wu et al. 2023; Ho et al. 2024; Gao et al. 2024). By collecting information on digestive health and physical activity patterns in a different post‐industrial society, the United States, we were able to understand the role exercise plays in IBS risk and situate this within an evolutionary framework to understand if lifestyle changes that reduce our exercise levels also have negative implications for our digestive health.

The sample Rome IV criteria IBS prevalence in Pennsylvania (28.77%) was higher than the reported nationwide average prevalence of 15%–20% (Altobelli et al. 2017). This is unsurprising, however, as many scholars agree that IBS is an underdiagnosed condition in the medical space, so using the criteria approach in a population sample likely provides a more accurate assessment of population IBS prevalence (Canavan et al. 2014; Drossman and Hasler 2016). The sample's physician diagnosis rate of 16.61% further reinforces this observation as it is clearly in line with diagnosed prevalence of IBS in the United States. For analyses, we utilized the Rome IV criteria, as it provided an accurate snapshot of present illness status and behavior while also aligning with discipline standards as Rome IV is the official diagnostic criteria. We also ran analyses using physician diagnosis, though of the variables analyzed the only significant association between IBS and physician diagnosis was female sex (*p* = 0.0003). This discrepancy between physician and Rome IV diagnosis in our sample may be due to more limited power for physician diagnosis or may instead reflect behavioral differences between those who have access to and utilize medical care and those who are experiencing symptoms but have not sought a physician diagnosis, either due to limited healthcare access or more moderate symptom severity.

While our study set out to understand if the mismatch between contemporary exercise levels and evolved energetic needs is associated with IBS by ascertaining the role of different exercise variables in IBS status, all exercise‐related predictors were insignificant, except for the vigor of exercise. Both model selection methods and post hoc analyses for discrimination and calibration reaffirmed these findings: physical activity is not a strong predictor for IBS status within this population. There was also no indication that exercise levels impacted specific demographics of the sample, such as women or individuals of a certain age.

One explanation for these findings may be due to insufficient power within the sample (*n* = 921). Although power calculations made prior to data collection suggested only needing a maximum of 306 individuals to detect an effect, post hoc power calculations (0.80 alpha and 0.05 beta) for exercise variables of interest indicated needing 4621 individuals for analyses of overall physical activity levels, 4361 individuals for analyses of time spent exercising, and 8653 individuals for analyses of exercise vigor. This is because there was much less variation in exercise patterns within the sample than anticipated during original power calculations.

An alternative, compelling theoretical explanation for these findings may be due to the differences between acute variables, such as physical activity per week, and longitudinal variables, like BMI. Physical activity levels can change significantly over the span of weeks or months, and a behavior that is so frequently modified may not be the strongest predictor in our correlation analysis, which would explain the low levels of association we found with these metrics. These findings suggest that IBS cannot be explained by a simple mismatch between current physical activity levels and evolved physical activity needs. Instead, the significant findings related to BMI as a predictor, as well as the fact that BMI is a clear mediator of the relationship between exercise vigor and IBS, are likely more effectively explained by the longitudinal effects of exercise on IBS, as proxied by BMI.

The use of BMI as a proxy for long‐term exercise behaviors allowed us to explore the potential role of energy mismatch in IBS. The relationship between BMI and exercise is not a direct one. Many people with higher BMIs are less likely to exercise, making BMI a potential proxy for exercise levels. Yet, it is important to also consider that a high BMI may be due to high muscle mass, caused by weight training or other forms of exercise. Investigating BMI across a variety of perspectives, including the interaction with CDC exercise recommendations, allows us to differentiate high muscle mass from obesity, and thus capture energy balance.

When BMI was added to our model, exercise vigor was no longer significant. BMI and exercise vigor were not collinear, and the interaction between BMI and vigor was also not significant, indicating that the relationship between exercise and disease status did not change substantially with different BMI levels. Ultimately, the mediation analysis suggests that BMI plays a significant mediating role in the relationship between exercise and disease status, over 22%, while exercise vigor plays a weak, inconsistent role in IBS risk. These findings support our hypothesis that IBS is driven by an energy imbalance due to mismatch and align with epidemiological research demonstrating that those experiencing obesity have a higher risk of IBS (Pickett‐Blakely 2014). Obesity, indicated by high BMI, was likely lower in the evolutionary past than it is now (Pontzer et al. 2012). Environmental mismatch, including mismatches in both exercise and diet (both changes in the types and quantity of food), contributes to an imbalance between energy intake and expenditure. These findings suggest that while exercise mismatch alone does not contribute to IBS, a larger environmental mismatch driven by post‐industrial behaviors is worth a more detailed investigation, particularly looking at nutrition and exercise jointly over time (Lenover and Shenk 2023; Pontzer et al. 2012).

Since this study was informed by other literature which did find significant results regarding physical activity and IBS (Fani et al. 2019; Hajizadeh Maleki et al. 2018; Yamamoto et al. 2023), the overall lack of association was surprising. Our study obtained the same variables collected in these previous studies, as well as additional measures of physical activity uncaptured in prior work. Yet, while other studies have identified associations, much of this work was completed within IBS patient cohorts, rather than in a population sample. In these studies, while sedentism was a common risk factor amongst IBS patients, there is no additional evidence demonstrating whether or how physical activity levels differed between IBS and non‐IBS patients. Additionally, much prior work was conducted in industrializing contexts, where there may be higher variation in levels of physical activity across the population. In contrast, our study aimed to determine the role of physical activity as an IBS risk factor within a post‐industrial population, with the finding that perhaps physical activity levels are not a strong predictor of IBS within this context.

We know that industrialization has changed our physical activity patterns, increasing sedentism and therefore the risk of many chronic diseases due to an energetic mismatch. This study suggests that IBS may not result primarily from mismatched physical activity alone, but instead, the long‐term effects of physical activity alongside other behavioral risk factors that contribute to energetic imbalances may play a significant role. For example, the significant role of BMI may actually reflect dietary components contributing to IBS, such as high‐fat foods or decreased fresh fruit and vegetable intake (Guo et al. 2015; Hosseini Oskouie et al. 2018; Na et al. 2021). The associations between BMI and IBS may be proximately explained by gut microbiome composition and/or brain‐gut axis regulation. Obesity is known to be linked to microbial dysbiosis, characterized by reduced diversity and an imbalance of bacterial species, which may contribute to IBS symptoms through increased gut permeability and low‐grade inflammation (Geng et al. 2022). Our previous research also identified several mismatch pathways by which IBS may originate, and in addition to diet and exercise, hygiene environments may play a key role in this relationship (Lenover and Shenk 2023). The Old Friends hypothesis, which hypothesizes the rise of illness alongside changes in environments (i.e., sterilization) has implications for a potential immune dysregulation pathway which may be intensified in urban areas (Rook et al. 2013). We know that physical activity patterns also vary in rural versus urban areas, and although we found no relationship between area of occupation, physical activity, and IBS (Table S4), we feel this is a valuable avenue for future investigation.

Additionally, we identified no dose–response relationship for exercise time or intensity, failing to support our hypothesis of an evolved maintenance range of physical activity in the past that is no longer met in current environments. This may be reflective of the variable nature of physical activity levels, indicating that a longitudinal approach would be better to capture these trends. Despite this, previous work which found associations between physical activity and IBS capturing physical activity at a singular moment in time like we did, may be demonstrating the importance of another behavioral element either highly correlated with exercise or mediating the relationship between IBS and exercise. Additionally, since exercise is known to improve stress levels, exercise may be contributing to a larger relationship between IBS and mental health, mediated by the brain‐gut axis (Lenover and Shenk 2023). Physical activity may regulate the brain‐gut axis by reducing stress‐related activation of the HPA axis and improving nervous system regulation, potentially reducing IBS symptoms (Fichna and Storr 2012).

In terms of limitations, our study design is subject to the common problems associated with all survey work, including the possibilities of biased recruitment and inaccurate recall. Recruiting via Prolific privileges those with internet access and solicits individuals who actively seek out surveys for compensation, rather than truly random participants, though Prolific has been demonstrated to be more effective than other platforms at mitigating these concerns (Douglas et al. 2023; Palan and Schitter 2018). Also, since the study was advertised as related to IBS, participants with stomach issues may have been more likely to respond, leading to a potentially inflated prevalence rate. In addition, in the context of exercise levels, this sample may also be on average less physically active than the broader population, given their participation in online research. Finally, since the data is cross‐sectional in nature, this study cannot directly test causation of IBS. Instead, our work offers methodologically sound assessments of association that allow us to test theoretical predictions and can provide promising target areas for future causal research.

In conclusion, within this Pennsylvania population, physical activity in isolation is not a significant risk factor for IBS, though BMI does play a key role in IBS risk and mediates the relationship between physical activity and IBS. These findings suggest that perhaps, rather than just a mismatch between our exercise needs and current‐day environments, there is a larger imbalance with the longitudinal effects of physical activity across the lifespan, impacting energetic intake and expenditure. While bias is possible due to our sampling methods, this study collects both novel variables, as well as existing variables via a more robust sampling protocol, making it a vital step to investigate relationships between exercise and IBS before launching into longitudinal work testing causation. Findings from this work may inform IBS prevention and treatment efforts, as well as continue to enrich our knowledge of the overall impact physical activity has—or does not have—on chronic disease.

## Author Contributions

**Makenna B. Lenover Moyer:** conceptualization, funding acquisition, investigation, writing – original draft, methodology, visualization, writing – review and editing, formal analysis, project administration. **Mary K. Shenk:** conceptualization, writing – review and editing, funding acquisition, resources, supervision, methodology.

## Conflicts of Interest

The authors declare no conflicts of interest.

## Supporting information


**Data S1:** ajpa70104‐sup‐0001‐Supinfo.docx.

## Data Availability

The data that support the findings of this study are openly available in Git Hub at https://github.com/mbl66/ibsexercise.

## Appendix I: Survey Instrument I

**Page 1: Screening**

*The following questions determine your eligibility in this study. Please answer honestly*.

1. Are you between the ages of 20 to 65?

a. Yes

b. No

2. Is English your primary language?

a. Yes

b. No

3. Do you reside in Pennsylvania?

a. Yes

b. No

4. Were you born in the United States?

a. Yes

b. No

5. Did you grow up in the United States since birth?

a. Yes

b. No

6. Are you willing to answer questions about your digestion and associated bowel movements?

a. Yes

b. No

**Page 2: Informed Consent**

**Page 3: Demographics**

*This section will ask about your demographic information, allowing us to better investigate how lifestyle influences IBS distribution within Pennsylvania. Please answer honestly, providing as much information as you feel comfortable with*.

1. What is your year of birth?

2. The area in which you currently reside is:

a. Rural

b. Suburban

c. Urban

3. What is your US zipcode?

4. What state did you grow up in?

5. For how long have you continuously lived in Pennsylvania?

a. Less than 1 year

b. 1–3 years

c. 4–7 years

d. 8–10 years

e. Greater than 10 years

f. My whole life

6. Which of the following best describes the total annual income of your current household from all sources?

a. Less than $20,000

b. $20,000 to $34,999

c. $35,000 to $49,999

d. $50,000 to $74,999

e. $75,000 to $99,999

f. $100,000 to $149,999

g. Over $150,000

7. How many people currently reside in your household?

Adults: (Drop down menu with numbers 0–10)

Children: (Drop down menu with numbers 0–10)

8. What is your occupation?

FILL IN

9. Which of the following best describes you? (select all that apply)

a. Asian or Pacific Islander

b. Black or African American

c. Hispanic or Latino

d. Native American or Alaskan Native

e. White or Caucasian

f. A race/ethnicity not listed here

10. What is your highest level of education?

a. Less than a high school diploma

b. High school degree or equivalent (GED)

c. Some college, no degree

d. Associates degree (e.g., AA, AS)

e. Bachelors degree (e.g., BA, BS)

f. Professional or trade certification (e.g., HVAC Technician, Cosmetology)

g. Masters degree (e.g., MA, MS, MBA)

h. Professional degree (e.g., JD, MD)

i. Doctorate degree (e.g., PhD, EdD)

11. What was your assigned sex at birth?

a. Female

b. Male

c. Other

12. With what gender do you identify?

a. Woman

b. Non‐binary

c. Man

d. Prefer to self‐describe, below

Self‐describe:

FILL IN

13. Are you currently taking any type of hormone replacement therapy medication (e.g., masculinizing hormone therapy, feminizing hormone therapy)?

a. Yes

b. No

If yes, what medication?

- Masculinizing hormone therapy
- Feminizing hormone therapy
- Other: FILL IN

14. Are you currently taking hormonal birth control (e.g., the pill, the ring, Norplant, injectable birth control)?

a. Yes

b. No

If yes, what medication?

- Combination Pill
- Mini pill (progestin only)
- Vaginal ring
- Injectable birth control
- Upper arm implant
- IUD
- Other: FILL IN

**Page 4: Irritable Bowel Syndrome Information**

*The following questions will provide insight on your digestive system, bowel movements, and potential IBS condition. Answering all the questions in this section is required for us to understand how digestive health relates to your lifestyle. If you do not feel comfortable answering any of these questions, the survey will end, though we thank you for your time participating in this study*.

*Questions 1 through 5 refer to your experience within the last 3 months*.

1. Have you experienced abdominal pain at least 1 day a week for the last 3 months?

a. Yes

b. No

If yes, how many days a week on average?

Drop down menu with numbers 1–7

2. Which of the following have you noticed in relation to abdominal pain? (select all that apply)

a. Change in frequency of bowel movements (either increasing or decreasing)

b. Pain improves or worsens with bowel movements

c. Change in bowel movement form/appearance

d. N/A I do not have abdominal pain

3. Does your abdominal pain change during/after defecation?

a. Yes, it increases

b. Yes, it decreases

b. No, it stays the same

c. N/A I do not have abdominal pain

4. On average how many times do you move your bowels?

a. 7+ times a day

b. 2–6 times a day

c. 1 time a day

d. 2–3 times a week

e. 1 time a week

5. In the past 3 months, have you had bloody stools?

- Yes
- No

6. Referencing this picture, how would you best characterize your bowel movements?

a. More than 25% of bowel movements like Types 1 and 2, and less than 25% like Types 6 and 7

b. More than 25% of bowel movements like Types 6 and 7, and less than 25% like Types 1 and 2

c. More than 25% of bowel movements like Types 6 and 7, and more than 25% of bowel movements like Types 1 and 2

d. None of the above

7. Regardless of whether you do or don’t currently, have you **ever** had abdominal pain at least once a week in the past for a period of 3 or more months?

a. Yes

b. No

8. In the past, did you experience any of the following alongside abdominal pain for a period of 3 or more months? (select all that apply)

a. Change in frequency of bowel movements (either increasing or decreasing)

b. Pain improves or worsens with bowel movements

c. Change in bowel movement form/appearance

d. N/A I did not have abdominal pain in the past

9. If you answered yes to question 7 and experienced symptoms in question 8, when did this occur?

a. Within the past year

b. 1 year ago

c. 2 years ago

d. 3 years ago

e. 4 years ago

f. 5 years ago

g. More than 5 years ago

10. If you answered yes to question 7 and experienced symptoms in question 8, for how long did this occur?

a. 3 months

b. 3–6 months

c. 6–9 months

d. 9–12 months

e. More than one year

11. Have you ever been diagnosed by a medical professional with IBS?

a. Yes

b. No

If yes, when?

- Within the past year
- 1 year ago
- 2 years ago
- 3 years ago
- 4 years ago
- 5 years ago
- More than 5 years ago

12. If yes, have you been diagnosed with:

a. IBS‐C (constipation)

b. IBS‐D (diarrhea)

c. IBS‐B (both constipation and diarrhea)

d. I do not know

13. Do you have a family history of IBS?

c Yes
d No

14. Who in your family has IBS (select all that may apply)

- Mother
- Father
- Sibling
- Other: Who?

15. Did you have colic as a child?

e Yes
f No
g Unsure

16. Have you ever been diagnosed with Crohn's disease, ulcerative colitis, or gastrointestinal cancer?

h Yes
i No

17. Have you been diagnosed with another digestive condition or illness? If so, select the illness(es) and the time of diagnosis below.

Illness listed with check box next to it, then also a drop‐down menu of time next to it

Celiac disease

SIBO (Small Intestine Bacterial Overgrowth)

Crohn's Disease

Ulcerative Colitis

Gastritis

Gastroesophageal reflux disease

Diverticulitis

Gastrointestinal Cancer

Other: FILL IN

TIME DROP DOWN MENU FOR EACH ILLNESS

Within the past year

1 year ago

2 years ago

3 years ago

4 years ago

5 years ago

More than 5 years ago

18. Have you been diagnosed with another condition or illness? If so, select the illness(es) and the time of diagnosis below.

Illness listed with check box next to it, then also a drop‐down menu of time next to it

Fibromyalgia

Chronic Migraines

Endometriosis

Chronic Fatigue

Chronic Pelvic Pain

Kidney Failure

Interstitial Cystitis

Dysuria

TIME DROP DOWN MENU FOR EACH ILLNESS

Within the past year

1 year ago

2 years ago

3 years ago

4 years ago

5 years ago

More than 5 years ago

**Page 6:** Exercise

*Physical activity and IBS are correlated. Both daily behaviors and exercise influence the condition. Please try to answer open and honestly to the best of your ability for each question*.

1. Some researchers think IBS and BMI (body mass index) are associated, but this relationship needs further investigation. To study this, we need your current height and weight to your best estimate.

Height (in feet and inches): FILL IN (2 boxes)

Weight (in pounds): FILL IN

2 How would you describe your overall average daily activity levels in the past 3 months?

- Sedentary: I get little to no movement, and spend most of my day seated (office work, driving, etc.)
- Moderately Active: I am sedentary for most of the day, but make sure my day involves movement (walking, standing desk, or a job that requires me on my feet, i.e., waiting tables, teaching, nursing)
- Vigorously Active: My job requires physical labor, or I spend at least 2 h a day doing vigorous exercise (agricultural worker, personal trainer, hotel housekeeper, etc.)
- Extremely Active: I am a training athlete, or my day fully involves intense physical activity (endurance, professional sport athlete, etc.)
- Other: Please Describe FILL IN

3. In the past 3 months, what types of activities do you do during your job and for how much time during your work week (select all that apply)?

– Sitting (e.g., office work, public transit driver)

NA/Don’t Do This Activity

25%

50%

75%

100%

– Standing (e.g., cashier, toll booth operator)

NA/Don’t Do This Activity

25%

50%

75%

100%

– Walking (e.g., waiter, dog walker)

NA/Don’t Do This Activity

25%

50%

75%

100%

– Heavy Lifting (e.g., construction, factory work)

NA/Don’t Do This Activity

25%

50%

75%

100%

– Endurance Exercise (e.g., running, physical training, swimming)

NA/Don’t Do This Activity

25%

50%

75%

100%

4. On average, in the past 3 months, other than for your job, how much time of additional exercise/physical activity do you get in a week?

a. 0 min

b. 0–75 min

c. 75–150 min

d. Over 150 min

5. On average, in the past 3 months, other than for your job, what vigor level of additional exercise/physical activity do you get in a week?

a. None

b. Mild

c. Moderate

d. High Intensity

6. On average, in the past 3 months, for how long do you participate in the following physical activities each week:

Walking

NA/I Do Not Do This Activity

– Less than 1 h
– 1 to 2 h
– 2 to 3 h
– 3 h plus

Endurance Exercise (e.g., running, swimming, cycling)

– NA/I Do Not Do This Activity
– Less than 1 h
– 1 to 2 h
– 2 to 3 h
– 3 h plus

Non‐Cardio Exercise Practices (e.g., yoga, Pilates, barre)

– NA/I Do Not Do This Activity
– Less than 1 h
– 1 to 2 h
– 2 to 3 h
– 3 h plus

Cardio Exercise Practices (e.g., dance, HIIT, cycling)

– NA/I Do Not Do This Activity
– Less than 1 h
– 1 to 2 h
– 2 to 3 h
– 3 h plus

Strength Training

– NA/I Do Not Do This Activity
– Less than 1 h
– 1 to 2 h
– 2 to 3 h
– 3 h plus
