# Supplementary material for: Physical Activity and Irritable Bowel Syndrome: The Role of Evolutionary Mismatch in Chronic Disease Risk
Source: Am J Biol Anthropol. 2025 Aug 1;187(4):e70104. doi: 10.1002/ajpa.70104 (PMC12315055; doi:10.1002/ajpa.70104)
Supplement: Supplementary file 1 — Data S1: ajpa70104‐sup‐0001‐Supinfo.docx. [file AJPA-187-e70104-s001.docx]

**SI Table 1:** Estimated sample sizes for variables of interest, using published prevalence rates.

| Variable of Interest | IBS Prevalence | % in IBS Group | % in Non-IBS Group | Reference | Estimated N | Study Country of Origin |
| --- | --- | --- | --- | --- | --- | --- |
| Physically Active (engaging in moderate to vigorous exercise once per week or more) | 19.1% | 86.5% | 60.2% | Chirila et al., 2012 | 82 | Romania |
| Engages in Regular Exercise (yes) | 15.8% | 8.5% | 19.5% | AlButaysh et al., 2020 | 306 | Saudi Arabia |

**SI Table 2:** Additional sample demographic and exercise frequency information for those diagnosed via the Rome IV criteria.

| Characteristic | Participants with IBS (n=265) | Participants without IBS (n=656) |
| --- | --- | --- |
| Income- n(%)  Less than $20,000  $20,000 to $34,999  $35,000 to $49,999  $50,000 to $74,999  $75,000 to $99,999  $100,000 to $149,999  Over $150,000 | 16 (6.04%)  28 (10.57%)  44 (16.60%)  60 (22.64%)  43 (16.23%)  48 (18.11%)  26 (9.81%) | 51 (7.77%)  64 (9.76%)  83 (12.65%)  141 (21.49%)  107 (16.31%)  123 (18.75%)  82 (12.50%) |
| Highest education- n(%)  Less than high school  High school degree/equivalent  Some college, no degree  Associates degree  Bachelors degree  Professional/trade certification  Masters degree  Professional degree  Doctorate degree | 0  49 (18.49%)  44 (16.60%)  31 (11.69%)  86 (32.45%)  4 (1.51%)  39 (14.72%)  5 (1.89%)  7 (2.64%) | 6 (0.91%)  86 (13.11%)  151 (23.02%)  66 (10.06%)  221 (33.69%)  5 (0.76%)  93 (14.18%)  15 (2.29%)  10 (1.52%) |

**SI Table 3:** Characteristics of study participants with self-reported, physician diagnosed IBS and healthy controls, including demographics and exercise frequency information.

| Characteristic | Participants with IBS (n=153) | Participants without IBS (n=768) |
| --- | --- | --- |
| Age (Mean +/- SD) | 40.56 +/- 11.88 | 38.42 +/- 11.91 |
| Sex (n, % Female) | 106 (69.28%) | 403 (52.47%) |
| IBS Subtype – n (%)  IBS-Diarrhea  IBS- Constipation  IBS Mixed | 29 (18.95%)  31 (20.26%)  38 (24.84%) | NA |
| Region of Residence- n (%)  Rural  Suburban  Urban | 38 (24.84%)  83 (54.25%)  32 (20.91%) | 171 (22.27%)  411 (53.52%)  179 (23.32%) |
| Race/ethnicity- n(%)  *Multiple selections allowed*  Asian or Pacific Islander  Black or African American  Hispanic or Latino  Native American or Alaskan Native  White or Caucasian | 0  21 (13.73%)  4 (2.61%)  1 (0.65%)  136 (88.89%) | 29 (3.78%)  108 (14.06%)  24 (3.13%)  4 (0.50%)  640 (83.33%) |
| Body Mass Index (BMI) (Mean +/- SD) | 30.00 +/- 8.50 | 28.12 +/- 7.51 |
| Income- n(%)  Less than $20,000  $20,000 to $34,999  $35,000 to $49,999  $50,000 to $74,999  $75,000 to $99,999  $100,000 to $149,999  Over $150,000 | 10 (6.54%)  12 (7.84%)  22 (14.38%)  47 (30.72%)  22 (15.68%)  24 (18.11%)  16 (10.46%) | 57 (7.42%)  80 (10.42%)  105 (13.67%)  154 (20.05%)  128 (16.66%)  147 (19.14%)  92 (11.98%) |

| Highest education- n(%)  Less than high school  High school degree/equivalent  Some college, no degree  Associates degree  Bachelors degree  Professional/trade certification  Masters degree  Professional degree  Doctorate degree | 1 (0.65%)  24 (15.69%)  22 (14.38%)  19 (12.42%)  50 (32.68%)  2 (1.31%)  27 (17.65%)  2 (1.31%)  6 (3.92%) | 5 (0.65%)  111 (14.45%)  173 (22.53%)  78 (10.16%)  257 (33.46%)  7 (0.91%)  105 (13.67%)  18 (2.34%)  11 (1.43%) |
| --- | --- | --- |
| Overall Sedentary Activity Level (n%) | 30 (19.61%) | 164 (21.35%) |
| 1 or Fewer Hours of Physical Activity Per Week (n%) | 32 (20.91%) | 170 (22.14%) |
| Mild or No Additional Exercise Per Week (n%) | 100 (64.10%) | 446 (58.07%) |

**SI Table 4:** Additional model outputs for demographic variables and controls in relation to Rome IV diagnosed IBS.

| Model | Sample Size | Coefficient Estimate | Odds Ratio | Confidence Interval | p-value | AIC |
| --- | --- | --- | --- | --- | --- | --- |
| IBS ~ Income Level  *Under $19,999*  $20,000 to $34,999  $35,000 to $49,999  $50,000 to $74,999  $75,000 to $99,999  $100,000 to $149,999  Over $150,000 | 67  92  127  201  150  171  108 | 0.33256  0.52459  0.30482  0.24761  0.01061  -0.0493 | 1.394  1.689  1.356  1.281  1.244  1.011 | 0.682-2.853  0.865-3.302  0.717-2.566  0.659-2.488  0.647-2.390  0.495-2.064 | 0.363  0.125  0.349  0.465  0.513  0.977 | 1112 |
| IBS ~ Region of Residence  *Rural*  Suburban  Urban | 209  494  211 | -0.04451  -0.05864 | 0.956  0.942 | 0.671-1.364  0.618-1.438 | 0.806  0.781 | 1105 |
| * (0.01 < p ≤0.05) **(0.001 < p ≤ 0.01) ***(p ≤ 0.001) | | | | | | |

**SI Table 5:** Model outputs for demographic variables and controls in relation to self-reported, physician diagnosed IBS.

| Model | Sample Size | Coefficient Estimate | Odds Ratio | Confidence Interval | p-value | AIC |
| --- | --- | --- | --- | --- | --- | --- |
| IBS ~ Age  Age | 906 | 0.007341 | 1.015 | 1.000-1.029 | 0.045* | 820 |
| IBS ~ Region of Residence  *Rural*  Suburban  Urban | 209  494  211 | -0.09568  -0.21757 | 0.909  0.804 | 0.595-1.388  0.481-1.346 | 0.658  0.408 | 831 |
| IBS ~ Income Level  *Under $19,999*  $20,000 to $34,999  $35,000 to $49,999  $50,000 to $74,999  $75,000 to $99,999  $100,000 to $149,999  Over $150,000 | 67  92  127  201  150  171  108 | -0.1566  0.1775  0.5537  -0.0205  0.0719  -0.0087 | 0.855  1.194  1.739  0.979  0.931  0.991 | 0.346-2.114  0.529-2.696  0.824-3.672  0.436-2.202  0.419-2.068  0.421-2.334 | 0.735  0.669  0.146  0.960  0.860  0.984 | 832 |
| IBS ~ Sex  *Male*  Female | 404  509 | 0.6921 | 1.998 | 1.543-3.247 | 0.0002 *** | 815 |
| IBS ~ Race  *Not White*  White | 144  776 | 0.4622 | 1.588 | 0.926-2.721 | 0.0928 | 829 |
| IBS ~ Age + Sex + Race  Age  Sex  Race | 899 | 0.0106  0.6778  0.4483 | 1.011  1.969  1.566 | 0.609-1.677  1.149-3.376  0.9133-2.684 | 0.160  0.0004***  0.120 | 805 |
| * (0.01 < p ≤0.05) **(0.001 < p ≤ 0.01) ***(p ≤ 0.001) | | | | | | |

**SI Table 6:** Model outputs for exercise and BMI variables and controls in relation to self-reported, physician diagnosed IBS.

| Model | Sample Size | Coefficient Estimate | Odds Ratio | 95% Confidence Interval | p-value | AIC |
| --- | --- | --- | --- | --- | --- | --- |
| IBS ~ Overall Physical Activity + Age + Sex + Race  *Vigorous/Extremely Active*  Sedentary  Moderately Active  Age  Sex  Race | 140  191  553 | 0.1671  0.2274  0.0111  -0.6665  0.4300 | 1.182  1.255  1.011  1.947  1.537 | 0.752-1.857  0.682-2.309  0.996-1.026  1.324-2.865  0.877-2.692 | 0.460  0.465  0.143  0.001***  0.133 | 800 |
| IBS ~ Over 75 Minutes of Activity a Week + Age + Sex + Race  *75 Minutes or More*  Under 75 Minutes  Age  Sex  Race | 589  308 | -0.1009  0.0099  0.6995  0.4204 | 0.904  1.010  2.013  1.522 | 0.621-1.316  0.995-1.025  1.375-2.947  0.869-2.669 | 0.598  0.184  0.0003***  0.142 | 803 |
| IBS ~ Over 150 Minutes of Activity a Week + Age + Sex + Race  *Over 150 Minutes*  Under 150 Minutes  Age  Sex  Race | 412  476 | -0.2222  0.0098  0.6624  0.4530 | 0.801  1.009  1.939  1.573 | 0.558-1.148  0.995-1.025  1.326-2.837  0.897-2.758 | 0.227  0.193  0.0001***  0.114 | 801 |
| IBS ~ Vigor of Activity + Age + Sex + Race  *Moderate/High Vigor*  None/Mild Vigor  Age  Sex  Race | 360  533 | 0.1658  0.0100  0.6566  0.4355 | 1.180  1.010  1.928  1.546 | 1.076-2.004  0.974-0.999  1.629-3.045  1.109-2.737 | 0.381  0.182  0.001***  0.127 | 804 |
| IBS ~ Meeting CDC Activity Levels + Age + Sex + Race  *Meeting Levels*  Not Meeting Levels  Age  Sex  Race | 336  553 | 0.2601  0.0097  20.6511  0.4259 | 1.297  1.009  1.918  1.531 | 0.886-1.899  0.995-1.025  1.309-2.808  0.874-2.681 | 0.181  0.200  0.001***  0.136 | 802 |
| IBS ~ BMI Continuous + Age + Sex + Race  BMI  Age  Sex  Race | 890 | -0.0134  0.0119  -0.6997  0.4082 | 0.987  1.012  2.012  1.504 | 0.964-1.010  0.997-1.027  1.377-2.945  0.858-2.636 | 0.269  0.117  0.0003***  0.154 | 802 |
| IBS ~ 3 Scale BMI + Sex + Age + Race  *Underweight/Healthy*  Overweight  Obese  Age  Sex  Race | 312  276  310 | 0.0909  -0.2009  0.0109  0.7046  0.4157 | 1.095  0.818  1.011  2.023  1.515 | 0.702-1.709  0.469-1.426  0.996-1.026  1.375-2.977  0.864-2.656 | 0.6888  0.4789  0.1576  0.0004***  0.1467 | 807 |
| * (0.01 < p ≤0.05) **(0.001 < p ≤ 0.01) ***(p ≤ 0.001) | | | | | | |
